# Relationship between angiotensin I-converting enzyme insertion/deletion gene polymorphism and retinal vein occlusion

**DOI:** 10.1186/1477-9560-12-17

**Published:** 2014-08-12

**Authors:** Işıl Kutluturk, Ali Karagöz, Tahir Bezgin, Vecih Oduncu, Ali Elveran, Cem Doğan, Ahmet Elbay, Cevat Kirma, Yusuf Özertürk

**Affiliations:** 1 Kartal Dr. Lütfi Kırdar Training & Research Hospital, Department of Ophthalmology, 34846 İstanbul, Turkey; 2 Kartal Kosuyolu Heart & Research Hospital, Department of Cardiology, Denizer Cad. Cevizli, Kartal-34846 İstanbul, Turkey; 3 Pendik State Hospital, Ophthalmology Clinic, İstanbul, Turkey

**Keywords:** Retinal vein occlusion, Angiotensin I-converting enzyme, Polymorphism

## Abstract

To evaluate the association between angiotensin I-converting enzyme insertion/deletion (ACE I/D) gene polymorphism and retinal vein occlusion (RVO). A total of 80 patients with retinal vein occlusion who was admitted to the Eye Department of Kartal Training and Research Hospital between 2008 and 2011, and 80 subjects were enrolled in this retrospective case–control study. Patients who experienced RVO within one week to six months of study enrolment were included, and those with coronary artery diseases, prior myocardial infarction history and coagulation disturbances were excluded from the study. The diagnosis was made by ophthalmoscopic fundus examination and fluorescein angiography. The ACE gene I/D polymorphism was determined by polymerase chain reaction, and the ACE gene was classified into three types: I/I, I/D and D/D. In multivariate logistic regression analysis, ACE D/D genotype (p = 0.035), diabetes-mellitus (p = 0.019) and hypertension (p = 0.001) were found to be independent predictive factors for RVO. The results of the present study reveal that ACE D/D polymorphism is an independent predictive factor for RVO. However, one cannot definitely conclude that ACE gene polymorphism is a risk factor for retinal vein occlusion.

## Introduction

Angiotensin I-converting enzyme (ACE), dipeptidyl peptidase, is a membrane-bound enzyme, which is present in endothelial and epithelial cells of various tissues, and innards including lungs and kidneys. Angiotensin I-converting enzyme converts Angiotensin I to Angiotensin II, a very potent vasoconstrictor agent [1]. Angiotensin II is a hormone as well as a locally produced cellular factor, directly affecting vascular endothelial cells and smooth muscles [2]. Furthermore, it has been demonstrated that receptors of Angiotensin II are found in the atherosclerotic vessel walls [3]. It is pointed out that Angiotensin II can promote vasoconstriction, inflammation and thrombosis in the vascular endothelium and vessel walls [4]. Besides being a potent vasoconstrictor, Angiotensin II is a proatherogenic agent, which elevates plasminogen activator inhibitor-1 (PAI-1) levels, which results in a decrease in the fibrinolytic activity [5,6].

Previous studies have reported that plasma levels of angiotensin II are closely associated with ACE insertion/deletion (I/D) polymorphism and that the serum level of ACE is likely to increase two-fold in the presence of ACE D/D polymorphism, consequently increasing the levels of plasma angiotensin II [7]. It has also been emphasized that the ACE I/D gene polymorphism might be an independent risk factor for thrombotic diseases [8-10].

There are very few studies examining the relationship between ACE gene polymorphism and retinal vein thrombosis, with controversial results [11-14]. Therefore, we aimed to evaluate the association between ACE I/D polymorphism and retinal vein occlusion (RVO).

## Methods

This case–control multi-center study composed of 80 patients, who experienced RVO one week to six months before enrolment. Control group composed of age and sex matched 80 persons in which retinal vein occlusion, other ocular diseases excluded with detailed ocular examination who referred to the eye clinic from internal medicine clinics in which study conducted. Patients gave written informed consent in accordance with the Declaration of Helsinki. The Institutional Review Board of Kartal Koşuyolu Heart Training and Research Hospital approved the study.

### Patients and controls

The patients with RVO and controls underwent a general physical examination, and a thorough cardiovascular and ophthalmic examination. A detailed medical history was taken from the study cohort. We excluded the subjects who had diabetic and/or hypertensive retinopathy findings among the controls. This is because subjects having vascular changing related to diabetes mellitus (DM) and/or hypertension (HT) might cause confusion while being evaluated the retinopathy related to RVO.

The diagnosis of RVO was made by ophthalmoscopic fundus examination and flourescein angiography. On the fundus examination, disc swelling, venous dilation or tortuosity, retinal hemorrhages, cotton wool spots and on the flourescein angiography demonstrating extensive areas of capillary closure, venous filling defects and increased venous transit time were assesed as the diagnosis of RVO by the same ophthalmologist.

The patients and controls were assessed for coagulation abnormalities and thrombosis (antithrombin III, protein C and protein S deficiency, lupus anticoagulant, anticardiolipin antibodies, activated protein C resistance, factor V Leiden mutation, prothrombin 20210 mutation, mean platelet volume, homocysteine, PAI-1 and lipoprotein A levels).Patients with abnormalities in coagulation parameters, previous thrombosis and family history of thrombosis, using oral contraceptives and hormone replacement therapy, having renal and coronary artery diseases and a prior history of myocardial infarction were excluded.

Hypercholesterolemia was defined as a total serum cholesterol level >200 mg/dL on admission or maintenance of normal cholesterol levels with statin therapy. Hypertension was defined as a systolic blood pressure ≥140 mmHg and a diastolic blood pressure ≥90 mmHg or current use of antihypertensive medications. Patients on oral anti-diabetic drug (OAD) therapy and/or insulin were considered as having diabetes.

## Detection of ACE polymorphisms

Polymerase chain reaction (PCR)–cDNA coagulation measures were performed to detect ACE polymorphisms in cases with RVO and in controls. Blood samples were taken from the antecubital vein after an overnight fasting. Whole blood samples from the patients were collected in ethylenediaminetetraacetic acid (EDTA) tubes. Total genomic DNA was isolated from whole blood samples using GenXtract DNA extraction system according to the manufacturer’s instructions (Vienna Lab Diagnostic GmbH). Then, target DNA regions were amplified by multiplex polymerase chain reaction (PCR) using biotinylated primers. Thereafter, amplified products were separated on 3% agarose gel. After observing the amplicons of the related genes, the amplified products were hybridized to a test strip containing allele specific nucleotide probes immobilized on a nitrocellulose membrane, using Cardiovascular Disease (CVD) Strip Assay (Vienna Lab Diagnostic GmbH). Hybridization process was performed with Tecan Profiblot T48 hybridization device. Bound biotinylated sequences were detected using streptavidin-alkaline phosphatase and color substrates.

Angiotensin I-converting enzyme gene was classified as I/I, I/D, D/D. The ACE gene I/D polymorphism was determined by PCR, using a primer pair flanking the polymorphic region of intron 16 that produces an amplified 490-bp (I allele), a 190-bp product (D allele) or both. All the reactions were performed according to the method by described previously [15]. The allele frequency was confirmed according to Hardy-Weinberg equilibrium (Table 1) [16].

**Table 1:** Hardy-Weinberg Equilibrium Result

**Genotype**	**Expected**	**Observed**
**Common homozygotes**	37.13	38
**Heterozygotes**	34.74	33
**Rare homozygotes**	8.13	9

*X2 =* 0.2 (80 samples counted) for likelihoods of calculated X2 value see above. p allele freq = 0.68; q allele freq = 0.32.

### Statistical analysis

Continuous variables were expressed as mean ± standard deviation. Categorical variables were expressed as percentages. The group means of continuous variables were compared using an independent samples t-test. Categorical variables were compared using the chi-square or the Fisher’s exact tests. Multivariate logistic regression analysis was applied to identify the independent predictors of RVO. Variables which identified as significant in the univariate analysis (diabetes mellitus, hypertension, hyperlipidemia, current smoking, ACE D/D and I/D polymorphism) were included in the model. Two-tailed p values <0.05 were considered to indicate statistical significance. The Statistical Package for the Social Sciences (SPSS, Inc., Chicago, IL, USA) version 11.5 was used for all statistical analyses.

## Results

This case–control study included 80 RVO patients (48.7% males) with a mean age of 60.2 ± 12.1 years, and 80 controls (47.5% males) with a mean age of 59 ± 12.6 years. Baseline demographic characteristics of the patients and control subjects are shown in Table 2. Frequency of hypertension (53.8 vs. 27.5%, p = 0.001), diabetes mellitus (38.8 vs. 15%, p = 0.001), hyperlipidemia (46.3 vs. 30.0%, p = 0.034 ) and current smoking (51.3 vs. 31.3%, p = 0.01) was significantly much higher among patients with RVO, when compared with that of controls. However, age, gender, BMI and family history for CAD were similar between the two groups (Table 2).

**Table 2:** Baseline demographic characteristics of the RVO patients and control subjects

	**RVO patients (n = 80)**	**Control (=80)**	**p**
**Age (year)**	60.2 ± 12.1	59 ± 12.6	0.54
**Female (%)**	41 (51.3)	42 (52.5)	0.87
**Hypertension (%)**	43 (53.8)	22 (27.5)	0.0001*
**Diabetes mellitus (%)**	31 (38.8)	12 (15.0)	0.001*
**Hyperlipidemia (%)**	37 (46.3)	24 (30.0)	0.034*
**Current smoking (%)**	41 (51.3)	25 (31.3)	0.01*
**History for CAD (%)**	23 (28.8)	17 (21.3)	0.27
**BMI (kg/m**^**2**^**)**	23.4 ± 3.1	23.8 ± 3.3	0.43

*p < 0.05.

Data are presented as mean ± standard deviation or number (%), where appropriate.

RVO, retinal vein occlusion; CAD, coronary artery disease; BMI, body mass index.

**Figure 1:**
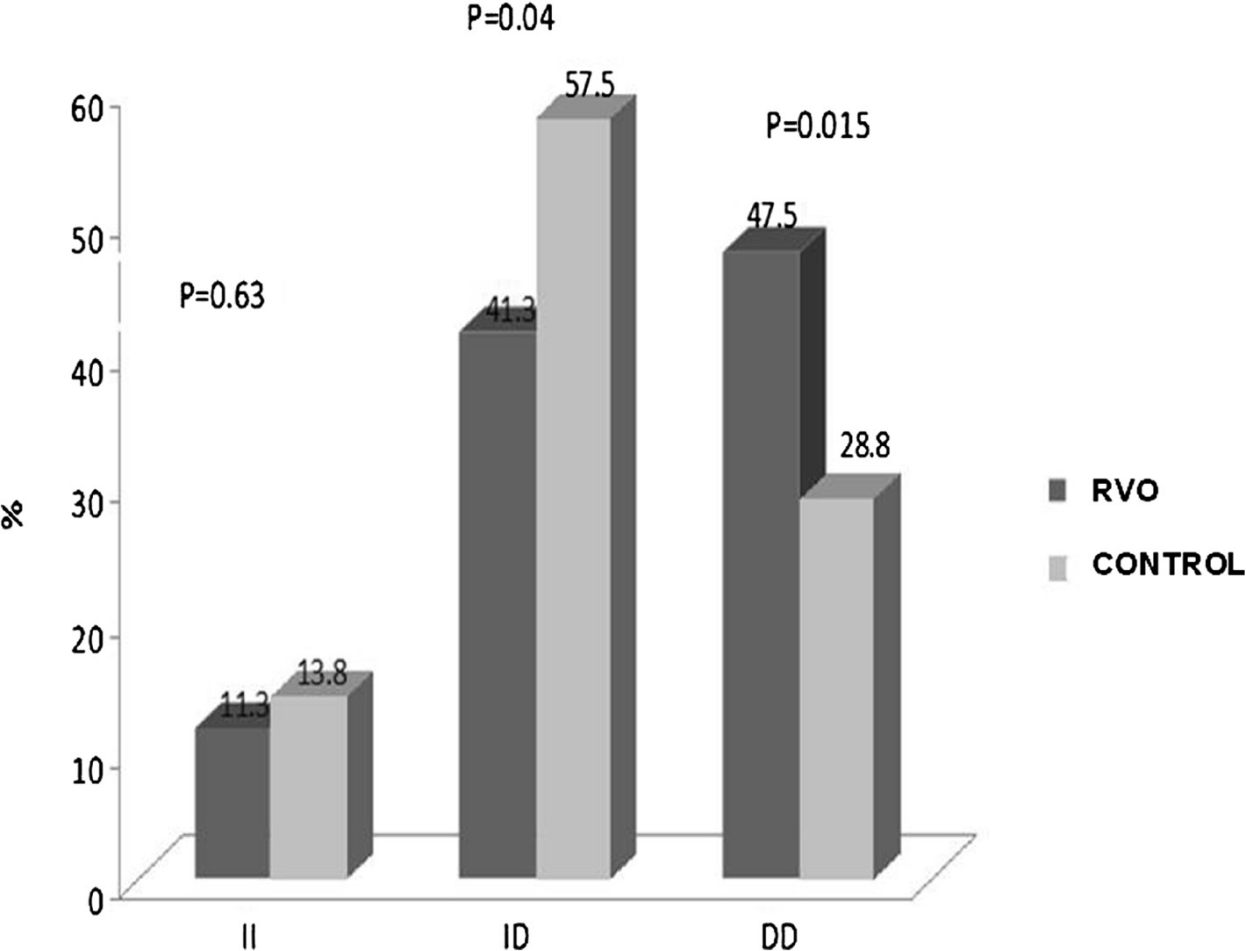
Comparison of frequencies of angiotensin I-converting enzyme I/D gene polymorphisms in patients with RVO vs. controls.

Angiotensin I-converting enzyme D/D gene polymorphism was significantly higher in patients with RVO (47.5 vs. 28.8%, p = 0.015), while ACE I/D polymorphism was significantly lower in patients with RVO than that of the controls (41.3 vs. 57.5%, p = 0.04). However, with regard to ACE I/I gene polymorphism, there was no significant difference between the two groups (11.3 vs. 13.8%, p = 0.63; Table 3, Figure 1).

**Table 3:** Angiotensin I-converting enzyme I/D gene polymorphism in patients with RVO and control subjects

**ACE Genotype**	**RVO**	**Control**	**P value**
**II, n (%)**	9 (11.3)	11 (13.8)	0.63
**ID, n (%)**	33 (41.3)	46 (57.5)	0.04
**DD, n (%)**	38 (47.5)	23 (28.8)	0.015

Frequency of ACE DD polymorphism in hypertensives of control group was significantly lower compared to that of patients with RVO ( 20.1 vs. 47.5%, p < 0.05), in contrast ACE I/D polymorphism was significantly higher in hypertensive controls than patients with RVO (65.8 vs. 41.3%, p < 0.05). With regard to ACE I/I gene polymorphism, there was no significant difference between the two groups (11.3 vs. 14.1%, p = NS).

Frequency of ACE DD polymorphism in diabetics of control group was significantly lower compared to that of patients with RVO (23.8 vs. 47.5%, p < 0.05). ACE I/D polymorphism was significantly higher in the diabetic controls than patients with RVO (62.4 vs. 41.3%, p < 0.05). However, with regard to ACE I/I gene polymorphism, there was no significant difference between the two groups (11.3 vs. 13.4%, p = NS).

Angiotensin I-converting enzyme D/D genotype [Odds Ratio (OR) = 3.83, 95% Confidence Interval (CI) = 1.10-13.37, p = 0.035, diabetes mellitus (OR = 2.90, 95% CI = 1.19-7.09, p = 0.019) and hypertension (OR = 4.48, 95% CI 1.86-10.83, p = 0.001) were found to be the independent predictive factors for RVO in multivariate logistic regression analysis.

Current smoking, hyperlipidemia and ACE I/D genotype were the significant variables in the univariate analysis; however, these parameters lost significance in multivariate analysis (Table 4).

**Table 4:** Independent risk factors in terms of RVO-multivariate analysis results

	**OR**	**95% CI**	**P value**
**ACE DD genotype**	3.83	1.10-13.37	0.035
**Diabetes mellitus**	2.90	1.19-7.09	0.019
**Hypertension**	4.48	1.86-10.83	0.001
**Smoking**	1.56	0.76-3.36	0.21
**Hyperlipidemia**	1.70	0.81-3.57	0.16
**ACE ID genotype**	0.62	0.18-2.01	0.44

OR = Odds ratio, CI = Confidence Interval.

## Discussion

The main finding of the present study is that ACE D/D gene polymorphism is an independent predictive factor for RVO. ACE I/D gene polymorphism was found to be significantly lower in patients with RVO, when compared with controls; however, ACE I/I polymorphism was similar between the two groups.

Retinal vein occlusion is the second most common retinal vascular disease after diabetic retinopathy in the elderly. It leads to serious visual loss and blindness. RVO is classified as central (CRVO) and branch RVO (BRVO) depending on the site of occlusion. The prevalence of BRVO (0.6 - 1.6%) is greater than that of CRVO (0.1 - 0.4%) [13,14]. The pathogenesis is multifactorial and poorly understood. It has been assumed that atherosclerotic vascular changes resulting in mechanical compression of vessel walls and venous stasis, as well as trophic changes in the endothelium, intima and media layers of the veins are the main factors in the etiology of RVO [17-19].

The potential risk factors related to RVO include age, smoking, diabetes, hypertension, hyperlipidemia and open-angle glaucoma [20-22]. In addition, compression of the vein at the arteriovenous (A/V) crossing, degenerative changes of the vessel wall and plasma abnormalities, including resistance to activated protein C, deficiencies of protein C, protein S, and antithrombin III, prothrombin gene mutation, anti-phospholipid antibodies and hyperhomocysteinemia may be the other risk factors related to RVO [23].

Angiotensin II promotes vasoconstriction, inflammation and thrombosis, and as a proatherogenic factor, decreases fibrinolytic activity via increasing PAI-1 levels. Previous studies have shown that the plasma level of angiotensin II is closely associated with ACE gene polymorphisms. ACE I/D gene polymorphism has been described as an independent risk factor for thrombotic diseases as well [8-10].

On the other hand, many earlier studies have associated ACE I/D gene polymorphism with diabetes mellitus and hypertension, and stated that the results are specific to the community studied, and ethnicity [24-27]. Therefore, in order to identify ACE I/D gene polymorphism as a predictor for RVO, ACE allele frequency in healthy individuals and ACE gene polymorphism frequency in patients with essential hypertension and type 2 diabetes mellitus should be considered in the population studied [28,29].

In our study, the ACE D/D gene polymorphisms, diabetes mellitus, hypertension, hyperlipidemia, and smoking were found to be significantly higher in the RVO group compared to controls. Gori et al. [11] investigated whether PAI 4G/5G and ACE I/D polymorphisms were independent risk factors for RVO and whether they were associated with elevated PAI-1 activity levels. They demonstrated similar findings to our results that ACE D/D genotype is a risk factor for RVO and confirmed the role of hypofibrinolysis, documented by high levels of PAI-1 activity, in patients with RVO. Yioti et al. [12] in a very recent study, evaluated the polymorphisms related to thrombophilia/hypofibrinolysis in a Greek population, and concluded that there might be an association between increased risk for RVO and ACE I/D, MTHFR C677T, PAI-1 4G/5G and factor V Leiden polymorphisms. Although Sottilotta et al. [13] reported hyperhomocysteinemia as a risk factor for RVO but C677T MTHFR do not exist any association with RVO in Italian population. In addition Glueck et al. [14] demonstrated that factor V mutation, high levels of heritable factor VIII, high homocysteine levels and low levels of antithrombin III are associated with CRVO as a familial thrombophlia. There were studies reporting findings those are opposite to our study results [30]. Furthermore in a recent study an association among RVO and ACE gen polymorphism was not demonstrated [31]. In agreement with previous studies, ACE D/D gene polymorphism, diabetes mellitus and hypertension were found to be the independent predictive factors for RVO. However, the association obtained between RVO and ACE I/D gene polymorphism might be due to the association between ACE I/D gene polymorphism and diabetes mellitus and/or hypertension. Therefore, in order to draw definite conclusions, it is necessary to identify the frequency of ACE gene polymorphism in patients with hypertension and type 2 diabetes mellitus nation-wide. Compared to previous studies only one genetic factor was determined as a risk factor for RVO and other genetic factors including factor V H1299R and V Leiden, β-fibrinogen G455A, PAI-1 4G/5G, ACE I/D, HPA1, prothrombin G20210A, factor XIII Val34Leu, MTHFR A1298C and C677T polymorphisms, activated protein C and S, antithrombin III deficiency, antiphosolipid and anticardiolipin antibodies, hyperhomocysteinemia may be possible other risk factors and it is necessary to investigate these genetic factors for RVO in large scale study groups .

As a result, in this study, the ACE D/D gene polymorphism was found to be an independent predictive factor for RVO. However, it is difficult to say that this polymorphism is a definite risk factor for RVO, due to the scarcity of large-scale studies evaluating the distribution of ACE I/D gene polymorphism in patients with diabetes mellitus and/or hypertension among Turkish population. Therefore, studies performed on the relationship between RVO and ACE gene polymorphism should take into consideration other systemic risk factors for RVO, keeping in mind that the results obtained would be specific to the population studied. This will yield more meaningful results that will help develop new prophylactic and therapeutic strategies for the diagnosis, treatment and follow-up of RVO.

## Competing interests

The authors declare that they have no competing interests.

## Authors' contribution

IK drafted the manuscript, involved in conception, design, acquisition, analysis and interpretation of data. AK participated in the design of the study and performed the statistical analysis. TB conceived of the study, and participated in its design and coordination and helped to draft the manuscript. VO performed the statistical analysis, drafted the manuscript. AE involved in conception and design, acquisition of data.CD analysis and interpretation of data.AE analysis and interpretation of data.CK involved in conception and design, acquisition of data.YÖ participated in its design and coordination and helped to draft the manuscript. All authors read and approved the final manuscript.
